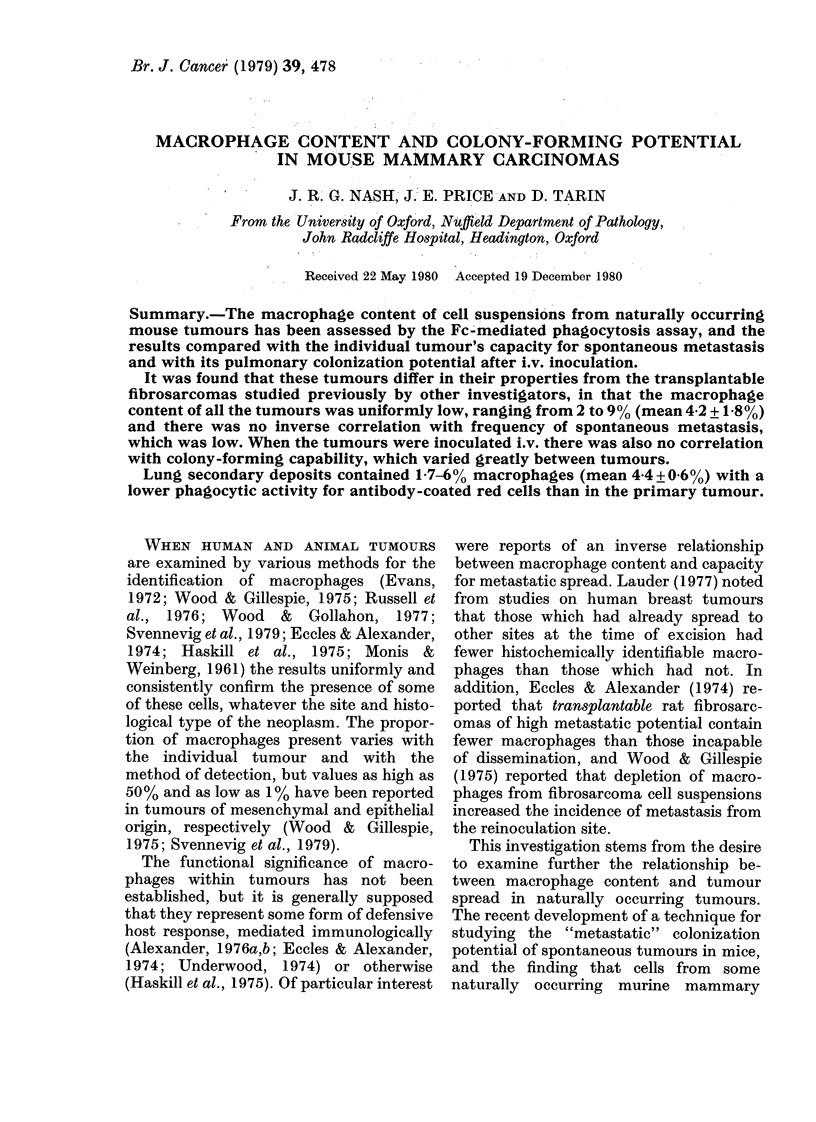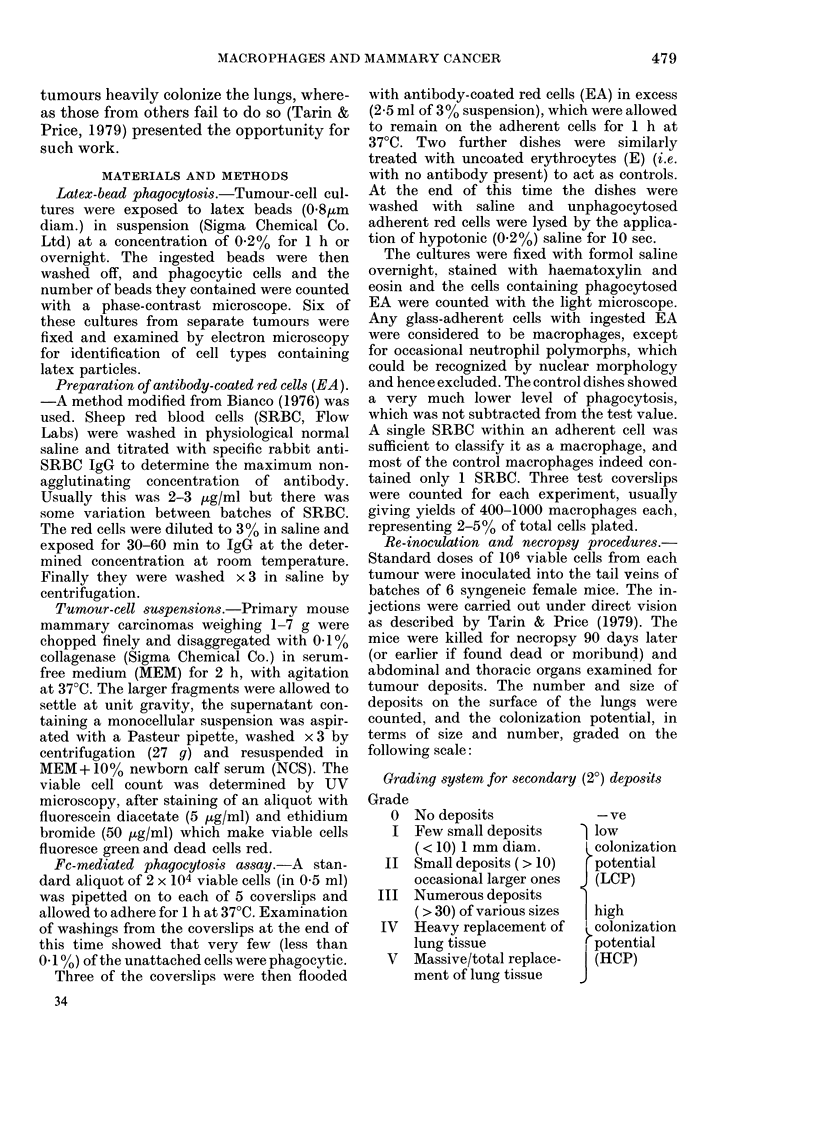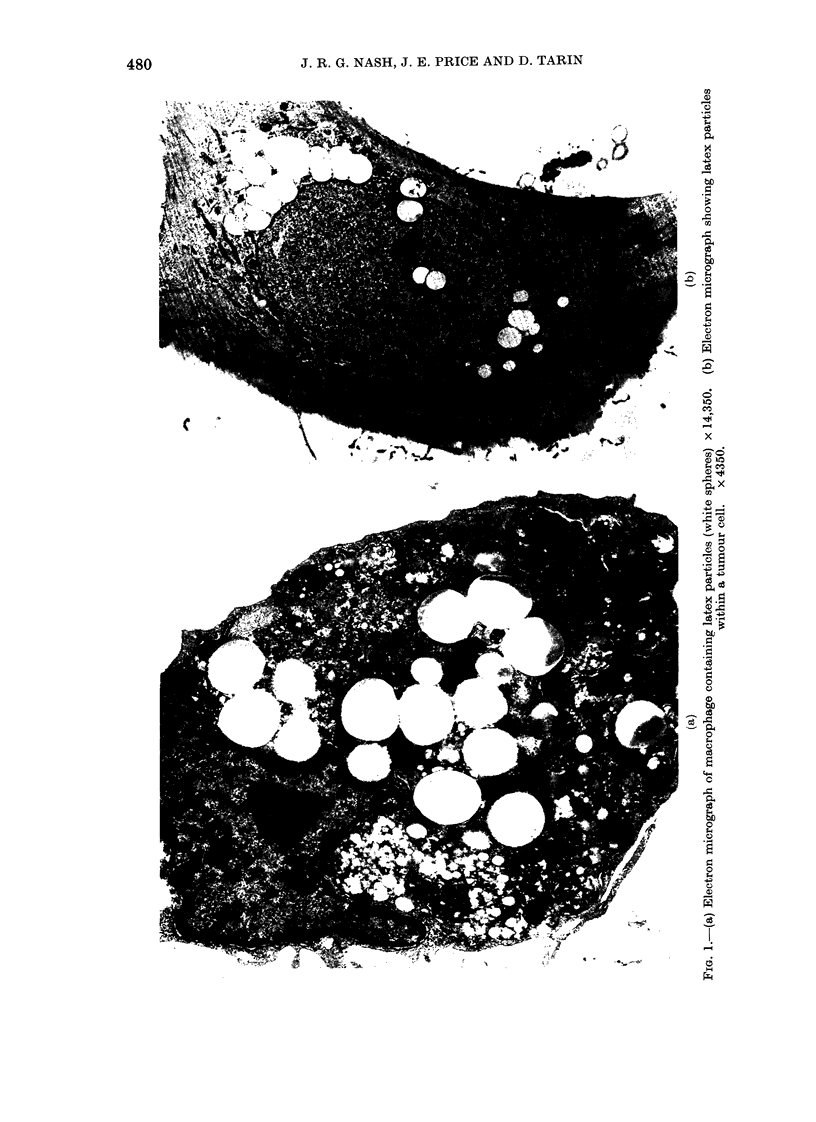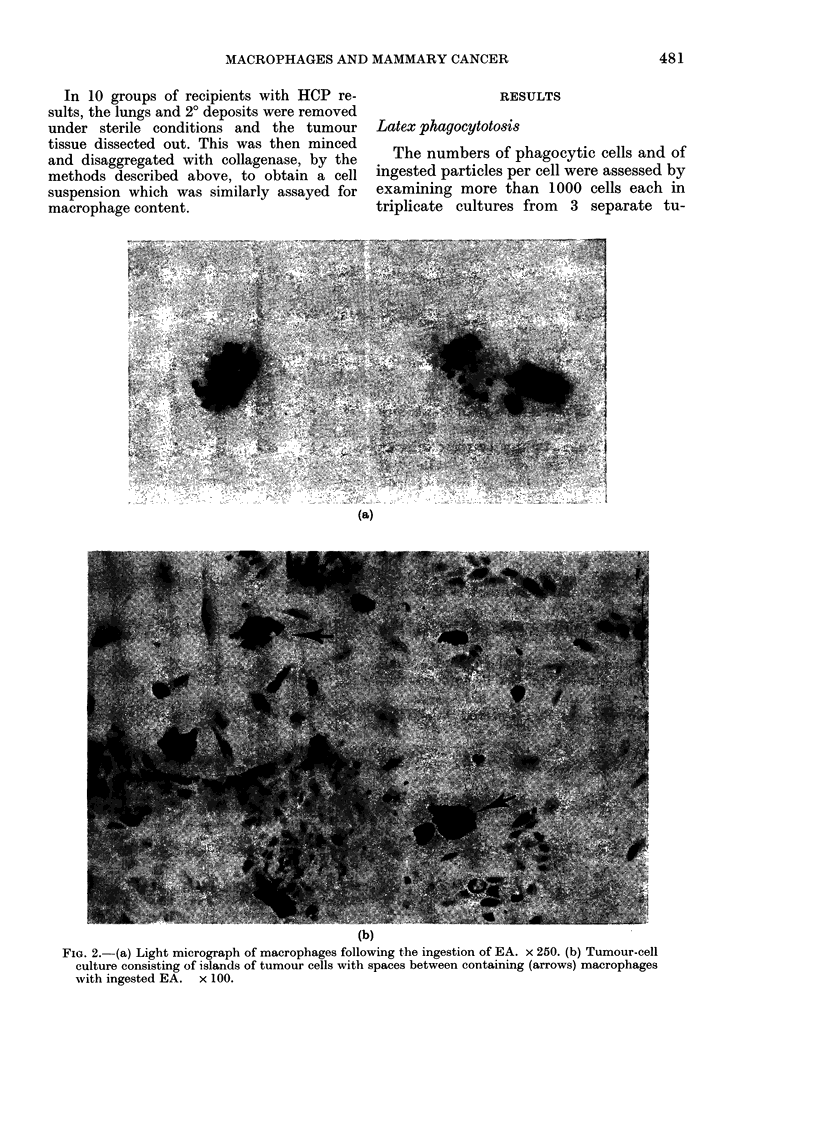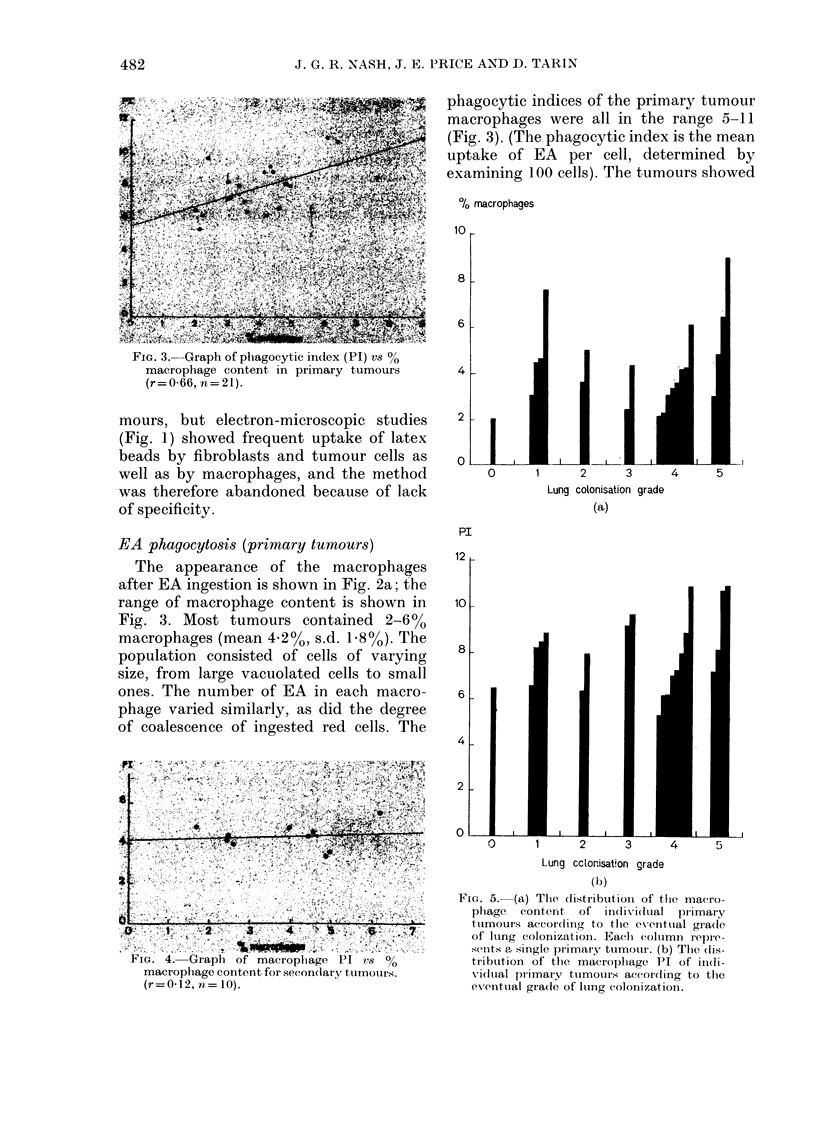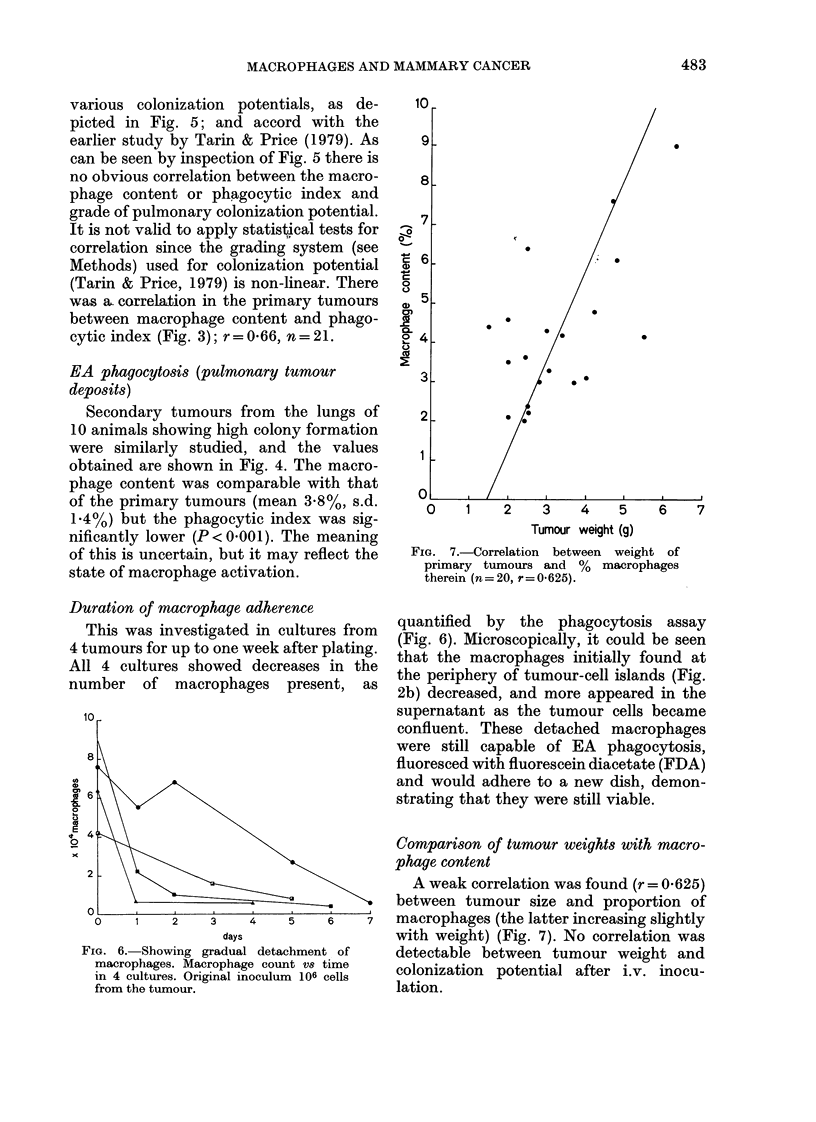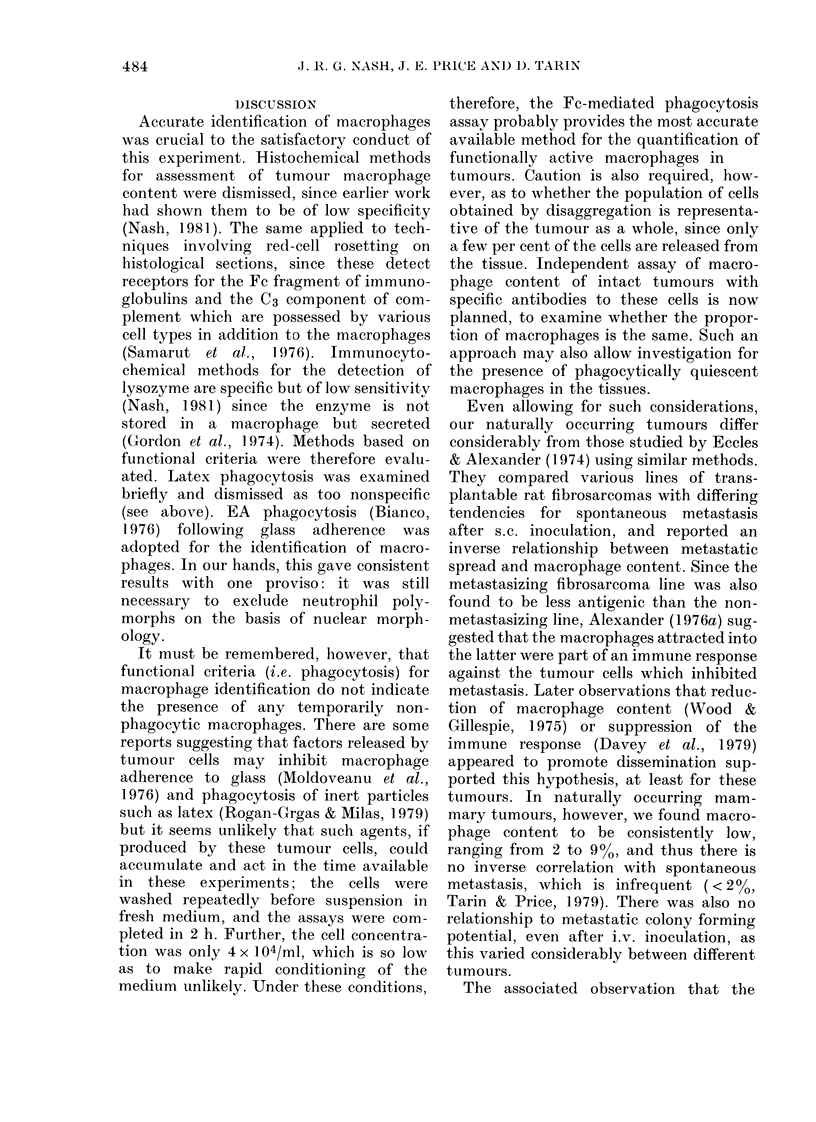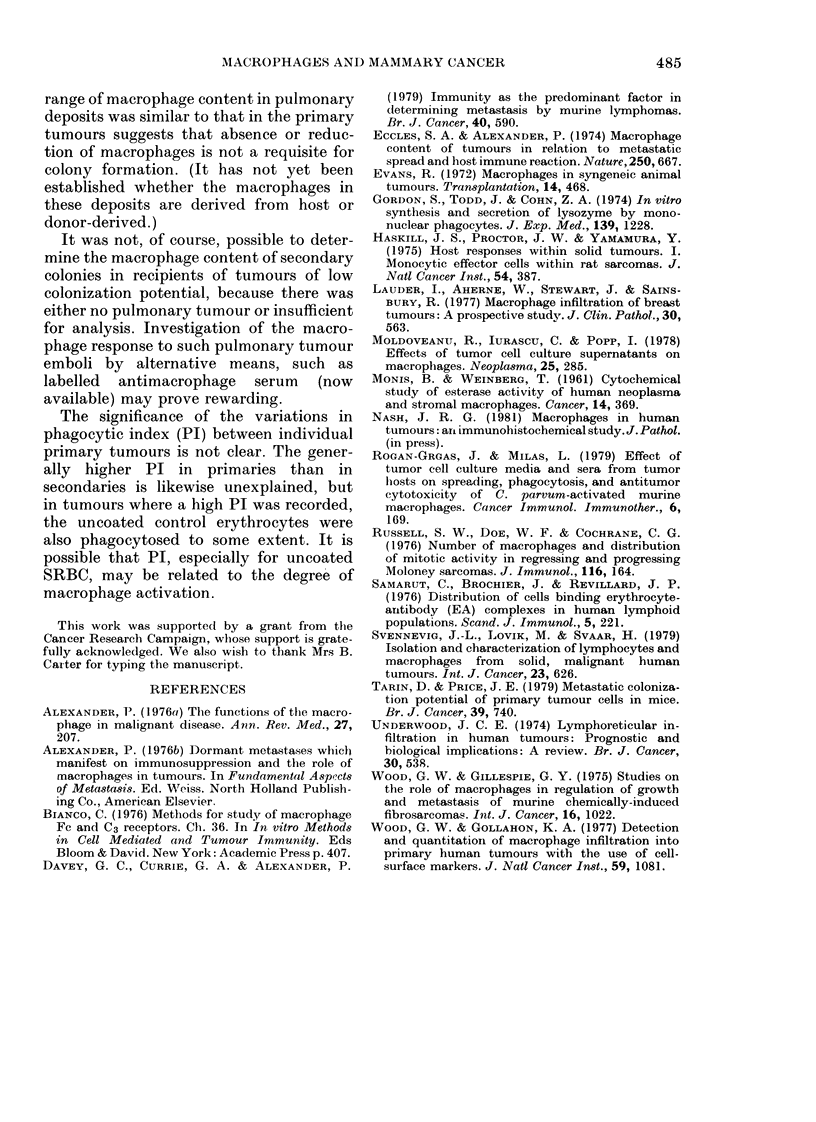# Macrophage content and colony-forming potential in mouse mammary carcinomas.

**DOI:** 10.1038/bjc.1981.70

**Published:** 1981-04

**Authors:** J. R. Nash, J. E. Price, D. Tarin

## Abstract

**Images:**


Br. J. Cancer (1979) 39, 478

### MACROPHAGE CONTENT AND COLONY-FORMING POTENTIAL IN MOUSE MAMMARY CARCINOMAS

J. R. G. NASH, J. E. PRICE AND D. TARIN

*From the University of Oxford, Nuffield Department of Pathology, John Radcliffe Hospital, Headington, Oxford*

Received 22 May 1980 Accepted 19 December 1980

**Summary.—The macrophage content of cell suspensions from naturally occurring mouse tumours has been assessed by the Fc-mediated phagocytosis assay, and the results compared with the individual tumour's capacity for spontaneous metastasis and with its pulmonary colonization potential after i.v. inoculation.**

**It was found that these tumours differ in their properties from the transplantable fibrosarcomas studied previously by other investigators, in that the macrophage content of all the tumours was uniformly low, ranging from 2 to 9% (mean 4·2 ± 1·8%) and there was no inverse correlation with frequency of spontaneous metastasis, which was low. When the tumours were inoculated i.v. there was also no correlation with colony-forming capability, which varied greatly between tumours.**

**Lung secondary deposits contained 1·7–6% macrophages (mean 4·4 ± 0·6%) with a lower phagocytic activity for antibody-coated red cells than in the primary tumour.**

When human and animal tumours are examined by various methods for the identification of macrophages (Evans, 1972; Wood & Gillespie, 1975; Russell *et al.*, 1976; Wood & Gollahon, 1977; Svennevig *et al.*, 1979; Eccles & Alexander, 1974; Haskill *et al.*, 1975; Monis & Weinberg, 1961) the results uniformly and consistently confirm the presence of some of these cells, whatever the site and histological type of the neoplasm. The proportion of macrophages present varies with the individual tumour and with the method of detection, but values as high as 50% and as low as 1% have been reported in tumours of mesenchymal and epithelial origin, respectively (Wood & Gillespie, 1975; Svennevig *et al.*, 1979).

The functional significance of macrophages within tumours has not been established, but it is generally supposed that they represent some form of defensive host response, mediated immunologically (Alexander, 1976*a*,*b*; Eccles & Alexander, 1974; Underwood, 1974) or otherwise (Haskill *et al.*, 1975). Of particular interest were reports of an inverse relationship between macrophage content and capacity for metastatic spread. Lauder (1977) noted from studies on human breast tumours that those which had already spread to other sites at the time of excision had fewer histochemically identifiable macrophages than those which had not. In addition, Eccles & Alexander (1974) reported that *transplantable* rat fibrosarcomas of high metastatic potential contain fewer macrophages than those incapable of dissemination, and Wood & Gillespie (1975) reported that depletion of macrophages from fibrosarcoma cell suspensions increased the incidence of metastasis from the reinoculation site.

This investigation stems from the desire to examine further the relationship between macrophage content and tumour spread in naturally occurring tumours. The recent development of a technique for studying the "metastatic" colonization potential of spontaneous tumours in mice, and the finding that cells from some naturally occurring murine mammary tumours heavily colonize the lungs, whereas those from others fail to do so (Tarin & Price, 1979) presented the opportunity for such work.

### MATERIALS AND METHODS

*Latex-bead phagocytosis.*—Tumour-cell cultures were exposed to latex beads (0·8$\mu$m diam.) in suspension (Sigma Chemical Co. Ltd) at a concentration of 0·2% for 1 h or overnight. The ingested beads were then washed off, and phagocytic cells and the number of beads they contained were counted with a phase-contrast microscope. Six of these cultures from separate tumours were fixed and examined by electron microscopy for identification of cell types containing latex particles.

*Preparation of antibody-coated red cells (EA).*—A method modified from Bianco (1976) was used. Sheep red blood cells (SRBC, Flow Labs) were washed in physiological normal saline and titrated with specific rabbit anti-SRBC IgG to determine the maximum non-agglutinating concentration of antibody. Usually this was 2–3 $\mu$g/ml but there was some variation between batches of SRBC. The red cells were diluted to 3% in saline and exposed for 30–60 min to IgG at the determined concentration at room temperature. Finally they were washed $\times 3$ in saline by centrifugation.

*Tumour-cell suspensions.*—Primary mouse mammary carcinomas weighing 1–7 g were chopped finely and disaggregated with 0·1% collagenase (Sigma Chemical Co.) in serum-free medium (MEM) for 2 h, with agitation at 37°C. The larger fragments were allowed to settle at unit gravity, the supernatant containing a monocellular suspension was aspirated with a Pasteur pipette, washed $\times 3$ by centrifugation (27 *g*) and resuspended in MEM + 10% newborn calf serum (NCS). The viable cell count was determined by UV microscopy, after staining of an aliquot with fluorescein diacetate (5 $\mu$g/ml) and ethidium bromide (50 $\mu$g/ml) which make viable cells fluoresce green and dead cells red.

*Fc-mediated phagocytosis assay.*—A standard aliquot of $2 \times 10^4$ viable cells (in 0·5 ml) was pipetted on to each of 5 coverslips and allowed to adhere for 1 h at 37°C. Examination of washings from the coverslips at the end of this time showed that very few (less than 0·1%) of the unattached cells were phagocytic.

Three of the coverslips were then flooded with antibody-coated red cells (EA) in excess (2·5 ml of 3% suspension), which were allowed to remain on the adherent cells for 1 h at 37°C. Two further dishes were similarly treated with uncoated erythrocytes (E) (*i.e.* with no antibody present) to act as controls. At the end of this time the dishes were washed with saline and unphagocytosed adherent red cells were lysed by the application of hypotonic (0·2%) saline for 10 sec.

The cultures were fixed with formol saline overnight, stained with haematoxylin and eosin and the cells containing phagocytosed EA were counted with the light microscope. Any glass-adherent cells with ingested EA were considered to be macrophages, except for occasional neutrophil polymorphs, which could be recognized by nuclear morphology and hence excluded. The control dishes showed a very much lower level of phagocytosis, which was not subtracted from the test value. A single SRBC within an adherent cell was sufficient to classify it as a macrophage, and most of the control macrophages indeed contained only 1 SRBC. Three test coverslips were counted for each experiment, usually giving yields of 400–1000 macrophages each, representing 2–5% of total cells plated.

*Re-inoculation and necropsy procedures.*—Standard doses of $10^6$ viable cells from each tumour were inoculated into the tail veins of batches of 6 syngeneic female mice. The injections were carried out under direct vision as described by Tarin & Price (1979). The mice were killed for necropsy 90 days later (or earlier if found dead or moribund) and abdominal and thoracic organs examined for tumour deposits. The number and size of deposits on the surface of the lungs were counted, and the colonization potential, in terms of size and number, graded on the following scale:

*Grading system for secondary* (2°) *deposits*

| Grade | | |
|---|---|---|
| 0 | No deposits | −ve |
| I | Few small deposits (< 10) 1 mm diam. | low colonization potential (LCP) |
| II | Small deposits (> 10) occasional larger ones | low colonization potential (LCP) |
| III | Numerous deposits (> 30) of various sizes | high colonization potential (HCP) |
| IV | Heavy replacement of lung tissue | high colonization potential (HCP) |
| V | Massive/total replacement of lung tissue | high colonization potential (HCP) |

FIG. 1.—(a) Electron micrograph of macrophage containing latex particles (white spheres) ×14,350. (b) Electron micrograph showing latex particles within a tumour cell. ×4350.

In 10 groups of recipients with HCP results, the lungs and 2° deposits were removed under sterile conditions and the tumour tissue dissected out. This was then minced and disaggregated with collagenase, by the methods described above, to obtain a cell suspension which was similarly assayed for macrophage content.

### RESULTS

### *Latex phagocytotosis*

The numbers of phagocytic cells and of ingested particles per cell were assessed by examining more than 1000 cells each in triplicate cultures from 3 separate tu-

FIG. 2.—(a) Light micrograph of macrophages following the ingestion of EA. ×250. (b) Tumour-cell culture consisting of islands of tumour cells with spaces between containing (arrows) macrophages with ingested EA. ×100.

FIG. 3.—Graph of phagocytic index (PI) *vs* % macrophage content in primary tumours ($r = 0{\cdot}66$, $n = 21$).

mours, but electron-microscopic studies (Fig. 1) showed frequent uptake of latex beads by fibroblasts and tumour cells as well as by macrophages, and the method was therefore abandoned because of lack of specificity.

*EA phagocytosis (primary tumours)*

The appearance of the macrophages after EA ingestion is shown in Fig. 2a; the range of macrophage content is shown in Fig. 3. Most tumours contained 2–6% macrophages (mean 4·2%, s.d. 1·8%). The population consisted of cells of varying size, from large vacuolated cells to small ones. The number of EA in each macrophage varied similarly, as did the degree of coalescence of ingested red cells. The phagocytic indices of the primary tumour macrophages were all in the range 5–11 (Fig. 3). (The phagocytic index is the mean uptake of EA per cell, determined by examining 100 cells). The tumours showed

FIG. 4.—Graph of macrophage PI *vs* % macrophage content for secondary tumours. ($r = 0{\cdot}12$, $n = 10$).

FIG. 5.—(a) The distribution of the macrophage content of individual primary tumours according to the eventual grade of lung colonization. Each column represents a single primary tumour. (b) The distribution of the macrophage PI of individual primary tumours according to the eventual grade of lung colonization.

various colonization potentials, as depicted in Fig. 5; and accord with the earlier study by Tarin & Price (1979). As can be seen by inspection of Fig. 5 there is no obvious correlation between the macrophage content or phagocytic index and grade of pulmonary colonization potential. It is not valid to apply statistical tests for correlation since the grading system (see Methods) used for colonization potential (Tarin & Price, 1979) is non-linear. There was a correlation in the primary tumours between macrophage content and phagocytic index (Fig. 3); $r = 0{\cdot}66$, $n = 21$.

*EA phagocytosis (pulmonary tumour deposits)*

Secondary tumours from the lungs of 10 animals showing high colony formation were similarly studied, and the values obtained are shown in Fig. 4. The macrophage content was comparable with that of the primary tumours (mean 3·8%, s.d. 1·4%) but the phagocytic index was significantly lower ($P < 0{\cdot}001$). The meaning of this is uncertain, but it may reflect the state of macrophage activation.

*Duration of macrophage adherence*

This was investigated in cultures from 4 tumours for up to one week after plating. All 4 cultures showed decreases in the number of macrophages present, as

FIG. 6.—Showing gradual detachment of macrophages. Macrophage count *vs* time in 4 cultures. Original inoculum $10^6$ cells from the tumour.

FIG. 7.—Correlation between weight of primary tumours and % macrophages therein ($n = 20$, $r = 0{\cdot}625$).

quantified by the phagocytosis assay (Fig. 6). Microscopically, it could be seen that the macrophages initially found at the periphery of tumour-cell islands (Fig. 2b) decreased, and more appeared in the supernatant as the tumour cells became confluent. These detached macrophages were still capable of EA phagocytosis, fluoresced with fluorescein diacetate (FDA) and would adhere to a new dish, demonstrating that they were still viable.

*Comparison of tumour weights with macrophage content*

A weak correlation was found ($r = 0{\cdot}625$) between tumour size and proportion of macrophages (the latter increasing slightly with weight) (Fig. 7). No correlation was detectable between tumour weight and colonization potential after i.v. inoculation.

### DISCUSSION

Accurate identification of macrophages was crucial to the satisfactory conduct of this experiment. Histochemical methods for assessment of tumour macrophage content were dismissed, since earlier work had shown them to be of low specificity (Nash, 1981). The same applied to techniques involving red-cell rosetting on histological sections, since these detect receptors for the Fc fragment of immunoglobulins and the $C_3$ component of complement which are possessed by various cell types in addition to the macrophages (Samarut *et al.*, 1976). Immunocytochemical methods for the detection of lysozyme are specific but of low sensitivity (Nash, 1981) since the enzyme is not stored in a macrophage but secreted (Gordon *et al.*, 1974). Methods based on functional criteria were therefore evaluated. Latex phagocytosis was examined briefly and dismissed as too nonspecific (see above). EA phagocytosis (Bianco, 1976) following glass adherence was adopted for the identification of macrophages. In our hands, this gave consistent results with one proviso: it was still necessary to exclude neutrophil polymorphs on the basis of nuclear morphology.

It must be remembered, however, that functional criteria (*i.e.* phagocytosis) for macrophage identification do not indicate the presence of any temporarily non-phagocytic macrophages. There are some reports suggesting that factors released by tumour cells may inhibit macrophage adherence to glass (Moldoveanu *et al.*, 1976) and phagocytosis of inert particles such as latex (Rogan-Grgas & Milas, 1979) but it seems unlikely that such agents, if produced by these tumour cells, could accumulate and act in the time available in these experiments; the cells were washed repeatedly before suspension in fresh medium, and the assays were completed in 2 h. Further, the cell concentration was only $4 \times 10^4$/ml, which is so low as to make rapid conditioning of the medium unlikely. Under these conditions, therefore, the Fc-mediated phagocytosis assay probably provides the most accurate available method for the quantification of functionally active macrophages in tumours. Caution is also required, however, as to whether the population of cells obtained by disaggregation is representative of the tumour as a whole, since only a few per cent of the cells are released from the tissue. Independent assay of macrophage content of intact tumours with specific antibodies to these cells is now planned, to examine whether the proportion of macrophages is the same. Such an approach may also allow investigation for the presence of phagocytically quiescent macrophages in the tissues.

Even allowing for such considerations, our naturally occurring tumours differ considerably from those studied by Eccles & Alexander (1974) using similar methods. They compared various lines of transplantable rat fibrosarcomas with differing tendencies for spontaneous metastasis after s.c. inoculation, and reported an inverse relationship between metastatic spread and macrophage content. Since the metastasizing fibrosarcoma line was also found to be less antigenic than the non-metastasizing line, Alexander (1976*a*) suggested that the macrophages attracted into the latter were part of an immune response against the tumour cells which inhibited metastasis. Later observations that reduction of macrophage content (Wood & Gillespie, 1975) or suppression of the immune response (Davey *et al.*, 1979) appeared to promote dissemination supported this hypothesis, at least for these tumours. In naturally occurring mammary tumours, however, we found macrophage content to be consistently low, ranging from 2 to 9%, and thus there is no inverse correlation with spontaneous metastasis, which is infrequent (< 2%, Tarin & Price, 1979). There was also no relationship to metastatic colony forming potential, even after i.v. inoculation, as this varied considerably between different tumours.

The associated observation that the range of macrophage content in pulmonary deposits was similar to that in the primary tumours suggests that absence or reduction of macrophages is not a requisite for colony formation. (It has not yet been established whether the macrophages in these deposits are derived from host or donor-derived.)

It was not, of course, possible to determine the macrophage content of secondary colonies in recipients of tumours of low colonization potential, because there was either no pulmonary tumour or insufficient for analysis. Investigation of the macrophage response to such pulmonary tumour emboli by alternative means, such as labelled antimacrophage serum (now available) may prove rewarding.

The significance of the variations in phagocytic index (PI) between individual primary tumours is not clear. The generally higher PI in primaries than in secondaries is likewise unexplained, but in tumours where a high PI was recorded, the uncoated control erythrocytes were also phagocytosed to some extent. It is possible that PI, especially for uncoated SRBC, may be related to the degree of macrophage activation.

This work was supported by a grant from the Cancer Research Campaign, whose support is gratefully acknowledged. We also wish to thank Mrs B. Carter for typing the manuscript.

### REFERENCES

ALEXANDER, P. (1976*a*) The functions of the macrophage in malignant disease. *Ann. Rev. Med.*, **27**, 207.

ALEXANDER, P. (1976*b*) Dormant metastases which manifest on immunosuppression and the role of macrophages in tumours. In *Fundamental Aspects of Metastasis*. Ed. Weiss. North Holland Publishing Co., American Elsevier.

BIANCO, C. (1976) Methods for study of macrophage Fc and $C_3$ receptors. Ch. 36. In *In vitro Methods in Cell Mediated and Tumour Immunity*. Eds Bloom & David. New York: Academic Press p. 407.

DAVEY, G. C., CURRIE, G. A. & ALEXANDER, P. (1979) Immunity as the predominant factor in determining metastasis by murine lymphomas. *Br. J. Cancer*, **40**, 590.

ECCLES, S. A. & ALEXANDER, P. (1974) Macrophage content of tumours in relation to metastatic spread and host immune reaction. *Nature*, **250**, 667.

EVANS, R. (1972) Macrophages in syngeneic animal tumours. *Transplantation*, **14**, 468.

GORDON, S., TODD, J. & COHN, Z. A. (1974) *In vitro* synthesis and secretion of lysozyme by mononuclear phagocytes. *J. Exp. Med.*, **139**, 1228.

HASKILL, J. S., PROCTOR, J. W. & YAMAMURA, Y. (1975) Host responses within solid tumours. I. Monocytic effector cells within rat sarcomas. *J. Natl Cancer Inst.*, **54**, 387.

LAUDER, I., AHERNE, W., STEWART, J. & SAINSBURY, R. (1977) Macrophage infiltration of breast tumours: A prospective study. *J. Clin. Pathol.*, **30**, 563.

MOLDOVEANU, R., IURASCU, C. & POPP, I. (1978) Effects of tumor cell culture supernatants on macrophages. *Neoplasma*, **25**, 285.

MONIS, B. & WEINBERG, T. (1961) Cytochemical study of esterase activity of human neoplasma and stromal macrophages. *Cancer*, **14**, 369.

NASH, J. R. G. (1981) Macrophages in human tumours: an immunohistochemical study. *J. Pathol.* (in press).

ROGAN-GRGAS, J. & MILAS, L. (1979) Effect of tumor cell culture media and sera from tumor hosts on spreading, phagocytosis, and antitumor cytotoxicity of *C. parvum*-activated murine macrophages. *Cancer Immunol. Immunother.*, **6**, 169.

RUSSELL, S. W., DOE, W. F. & COCHRANE, C. G. (1976) Number of macrophages and distribution of mitotic activity in regressing and progressing Moloney sarcomas. *J. Immunol.*, **116**, 164.

SAMARUT, C., BROCHIER, J. & REVILLARD, J. P. (1976) Distribution of cells binding erythrocyte-antibody (EA) complexes in human lymphoid populations. *Scand. J. Immunol.*, **5**, 221.

SVENNEVIG, J.-L., LOVIK, M. & SVAAR, H. (1979) Isolation and characterization of lymphocytes and macrophages from solid, malignant human tumours. *Int. J. Cancer*, **23**, 626.

TARIN, D. & PRICE, J. E. (1979) Metastatic colonization potential of primary tumour cells in mice. *Br. J. Cancer*, **39**, 740.

UNDERWOOD, J. C. E. (1974) Lymphoreticular infiltration in human tumours: Prognostic and biological implications: A review. *Br. J. Cancer*, **30**, 538.

WOOD, G. W. & GILLESPIE, G. Y. (1975) Studies on the role of macrophages in regulation of growth and metastasis of murine chemically-induced fibrosarcomas. *Int. J. Cancer*, **16**, 1022.

WOOD, G. W. & GOLLAHON, K. A. (1977) Detection and quantitation of macrophage infiltration into primary human tumours with the use of cell-surface markers. *J. Natl Cancer Inst.*, **59**, 1081.